# Assessment of the pulmonary adaptive immune response to *Cladosporium cladosporioides* infection using an experimental mouse model

**DOI:** 10.1038/s41598-020-79642-y

**Published:** 2021-01-13

**Authors:** Xiaoping Ma, Jing Hu, Yan Yu, Chengdong Wang, Yu Gu, Sanjie Cao, Xiaobo Huang, Yiping Wen, Qin Zhao, Rui Wu, Zhicai Zuo, Junliang Deng, Zhihua Ren, Shumin Yu, Liuhong Shen, Zhijun Zhong, Guangneng Peng

**Affiliations:** 1 Key Laboratory of Animal Disease and Human Health of Sichuan Province, College of Veterinary Medicine, Sichuan Agricultural University, Chengdu, 611130 China; 2 China Conservation and Research Center for the Giant Panda, Chengdu, 611800 Sichuan China; 3 College of Life Sciences, Sichuan Agricultural University, Chengdu, 611130 China

**Keywords:** Fungal host response, Fungal pathogenesis, Microbiology

## Abstract

*Cladosporium cladosporioides* causes asthma and superficial and deep infections, mostly in immunodeficient individuals and animals. This study aimed to investigate whether *C. cladosporioides* spores can enter the lungs through pulmonary circulation and influence pulmonary immune response. We intravenously injected mice with *C. cladosporioides* spore suspension and conducted several assays on the lungs. Pulmonary hemorrhage symptoms and congestion were most severe on days 1, 2, and 3 post-inoculation (PI). Extensive inflammatory cell infiltration occurred throughout the period of infection. More spores and hyphae colonizing the lungs were detected on days 1, 2, and 3 PI, and fewer spores and hyphae were observed within 21 d of infection. Numerous macrophages, dendritic cells, and neutrophils were observed on day 5 PI, along with upregulation of CD54, an intercellular adhesion molecule. Th1 and Th2 cells increased after infection; specifically, Th2 cells increased considerably on day 5 PI. These results suggest that days 2 and 5 PI represent the inflammatory peak in the lungs and that the Th2 and Th1 signaling pathways are potentially involved in pulmonary immune responses. In conclusion, the further adaptive immune responses played important roles in establishing effective pulmonary immunity against *C. cladosporioides* systemic infections based on innate immune responses.

## Introduction

*Cladosporium cladosporioides* is a common mold in both indoor and outdoor environments^1,2^. This species of dematiaceous hyphomycetes can infect immunocompetent and immunodeficient humans and animals and cause diseases including hemorrhagic pneumonia^3^, pulmonary phaeohyphomycosis^4^, and cutaneous phaeohyphomycosis^5–7^. This fungus has also been identified in vaginal samples from healthy giant pandas^8^. *C. cladosporioides* is an opportunistic fungal pathogen that infects hosts through three specific mechanisms: direct infection, dysregulation of immune responses, and toxic effects of fungal secondary metabolites^9^. However, *C. cladosporioides* spores in the air pose the risk of airway infections, such as hemorrhagic pneumonia^3^, and serve as allergens and infect the lungs, symptoms of which include asthma and allergic pneumonia^10^. Moreover, both spores and hyphae contain the same sensitizing factor; however, the total concentration of allergens in spores is higher than that in hyphae^11,12^, primarily due to specific allergens spores, rather than the total fungal load, exacerbating the allergic symptoms^13^.

The identification and elimination of fungal pathogens primarily depend on immune phagocytes, especially macrophages, neutrophils, and dendritic cells, which phagocytose fungal cells, initiate antigen presentation, and synthesize anti-microbial effector molecules and reactive nitrogen species^14^. Innate immune cells promote the adaptive immune response to produce inflammatory mediators, including chemokines and cytokines^15^, which generally take up fungal cells, inhibit fungal growth, and trigger acute inflammatory responses or present fungal antigens to T cells^16^. The stimulation of antigens and the release of pro-inflammatory cytokines are important to activate effective innate host defense and subsequently induce adaptive immune responses to eliminate pathogens completely.

Antigen-stimulated naive CD4^+^ T cells differentiate into functionally distinct subsets: Th1, Th2, Th17, regulatory T cells (Tregs), Th9, Th22, follicular Th cells (Tfh), and follicular Tregs effector cells^17^. Th1 cells primarily produce IFN-γ and TNF-α, which are key defenses against intracellular bacteria and viruses in the host, and participate in autoimmune disorders. Th2 cells primarily produce IL-4, IL-5, and IL-13, control helminth infections, and participate in allergic immune responses. Th17 cells secrete various cytokines, including IL-17A, IL-17F, IL-21, IL-22, and GM-CSF, and are primarily present at mucosal sites, including the gastrointestinal tract and airways. Furthermore, Tfh cells differentiate into IL-4/IL-13 double-producing Th2 cells that accumulate in the lungs and recruit eosinophils during allergic airway inflammation^18^.

β-glucan accounts for 60% of the cell wall and is critical for identifying molds via the immune system^19^. Dectin-1 helps recognize β-glucan at the surface of fungal cells; however, some fungal strains evade this recognition through agonist masking, as reported with infections by *Candida albicans*^20^ and *Aspergillus fumigates*^21^. Dectin-1 serves as a pattern recognition receptor (PRR) for β-glucan expressed in macrophages, monocytes, neutrophils, and dendritic cells^22^. Host immune responses against *C. cladosporioides* are mediated via effective recognition of surface β-glucan rather than total β-glucan levels in the fungal cell wall^23^. The dissolved form of β-glucan lacks stimulatory activity; however, β-glucan microparticles induce numerous cellular responses, including the production of cytokines and chemokines, which promote the development of innate and adaptive immune responses^24^. Toll-like receptors (TLRs), a type of important PRR, initiate innate and adaptive immune responses, and TLR-2/ TLR-4 recruit and amplify Tregs to regulate immune responses, thus maintaining immune homeostasis^25^. TLR-2/4 plays essential roles in fungal infections in association with carbohydrates generally, including mannan^26^.

In an asthma model intratracheally induced by molds, *C. cladosporioides* induced eosinophilia and a strong Th2 cellular response, while *A. versicolor* induced a strong Th17 cellular response and neutrophilic inflammation^27^. In another asthma model intratracheally induced with *C. cladosporioides*, the immune responses induced by live fungi appeared as strong Th2 immune responses and eosinophilia, while immune responses induced by heat-killed fungi appeared as strong Th17 immune responses and neutrophilic inflammation^23^.

*C. cladosporioides* has been isolated from skin lesions in the giant panda, and this strain causes phaeohyphomycosis, including surface and deep-tissue infection, and induces asthma owing to fungal allergenicity through different routes of infection. This study aimed to investigate whether *C. cladosporioides* enters the lungs through blood circulation, as well as the pathological symptoms and adaptive immune responses induced in the lungs after infection. We intravenously injected mice with *C. cladosporioides* spores and carried out a histopathological examination and a colony-forming unit (CFU) assay. Furthermore, immune cells recruited to the lungs were identified, and Th cell percentage and cytokine gene expression induced in the lungs of infected mice were assessed.

## Materials and methods

### Fungal strain

*C. cladosporioides* was originally isolated from the site of dermatosis in a giant panda and identified as a pathogen in skin lesions at the China Conservation and Research Center for the Giant Panda (Ya'an, Sichuan).

### Animals and experimental infection

Specific-pathogen-free 2-month-old female C57BL/6 mice were used herein. All animal experiments were approved by the Institutional Animal Care and Use Committee of Sichuan Agricultural University (permit number DYY-S20161108). *C. cladosporioides* was inoculated on an SDA medium and incubated in a microbiological incubator at 25 ℃ for 4 weeks. Thereafter, the fungi were scraped with sterile PBS from the SDA medium and transferred to a sterile centrifuge tube, and allowed to stand for 30 min. The fungal spores were concentrated in the middle liquid. Mice in the test groups were infected with 2 $\times$ 10^7^ spores/mL of *C. cladosporioides* spores suspended in sterile PBS. Experimental infection was induced intravenously, injecting the inoculums (200 μL) into the tail vein after light anesthesia with diethyl ether. Mice in the control group were intravenously administered sterile PBS (200 μL). For histological examination (3 mice per group), CFU assay (8 mice per group), immunofluorescence staining (3 mice per group) , quantitative RT-PCR (qRT-PCR)-based quantification of PRR and cytokine mRNAs (8 mice per group) , and flow cytometry (6 mice per group) , mice were randomly selected from each group. Mice in the control and test groups were euthanized via cervical dislocation, and tissue samples were harvested^28^.

### Histopathological analysis

Lung specimens were harvested on days 1, 2, 3, 5, 9, 14, and 21 post-inoculation (PI) and fixed with 10% neutralized buffered formalin and then stained with hematoxylin and eosin (H&E) for histopathological evaluation or periodic acid-Schiff (PAS) stain to assess fungal colonization in the lungs.

### CFU assay

Lung tissue specimens were harvested on days 2, 5, 9, and 14 PI and homogenized in PBS, and aliquots of the final suspensions were plated on SDA media and incubated at 25 ℃ for 5–7 d, and the numbers of recovered colonies were determined. The results were compared with those of the control group receiving intravenous sterile PBS and expressed as log-transformed CFU/g values for the organ.

### Immunofluorescence staining

On days 0 and 5 PI, lung specimens were harvested from mice and stained with species-specific antibodies for neutrophils, dendritic cells, macrophages, and intercellular adhesion molecule CD54, as previously reported^29–32^. The characteristics of the primary and secondary antibodies are listed in Table 1.

**Table 1:** Antibodies used for immunofluorescence staining.

Primary antibody	Target	Secondary antibody	Source
Rat antimouse CD54 (ICAM-I) biotin	CD54	Goat Anti-rat CY3	eBioscience (San Diego, CA, U.S.A.)
Hamster antimouse CD11c biotin	DCsand Macrophages	Goat Anti-rat CY3	eBioscience
Rat antimouse neutrophil	PMNs	Goat Anti-rat CY3	Abcam (Cambridge, U.K.)

DCs, dendritic cells; ICAM, intercellular adhesion molecule; PMNs, polymorphonuclear neutrophils.

### RNA isolation and qRT-PCR analysis

On days 0, 2, 5, 9, and 14 PI, lung biopsy specimens were homogenized with liquid nitrogen, using small porcelain mortars. Total RNA was isolated using TRIzol reagent in accordance with the manufacturer’s instructions (Invitrogen, Burlington, ON, Canada). The cDNA template was synthesized from RNA via reverse transcription, using Prime Script RT Reagent Kit with gDNA Eraser (TaKaRa, Kusatsu, Japan). qRT-PCR was carried out using TB Green Premix EX Taq II (TliRNaseH Plus) as a ready-to-use reaction mixture (TaKaRa, Kusatsu, Japan); the two-step method was used, including 2 min for 42 ℃ and 15 min for 37 ℃ with different reagents. Relative gene expression levels were normalized to that of hypoxanthine–guanine phosphoribosyltransferase (*Hgprt*). The cDNA was amplified using primers specific for cytokines and PRRs. The results in terms of cycle thresholds were converted to expression fold change values relative to *Hgprt* using the 2^–△△Ct^ method. SPSS20.0 software was used to evaluate the statistical differences between day 0 and other days. The primers for mouse *Hgprt* (internal control), PRRs, and cytokines are listed in Table 2.

**Table 2:** Sequences of oligonucleotide primers used for quantitative RT-PCR analysis.

Target gene	Primer sequence (5′ > 3′)	PCR product size (bp)
HGPRT	Forward: TGCCGAGGATTTGGAAAAAG Reverse: TGCCGAGGATTTGGAAAAAG	164
IL-6	Forward: GTTCTCTGGGAAATCGTGGA Reverse: TGTACTCCAGGTAGCTATGG	208
IL-1β	Forward: TTGACGGACCCCAAAAGATG Reverse: AGAAGGTGCTCATGTCCTCA	204
TNF-α	Forward: TCTCATCAGTTCTATGGCCC Reverse: GGGAGTAGACAAGGTACAAC	164
IL-22	Forward: GGCCAGCCTTGCAGATAACA Reverse: GCTGATGTGACAGGAGCTGA	220
IL-10	Forward: AGCCGGGAAGACAATAACTG Reverse: CATTTCCGATAAGGCTTGG	189
TFG-β	Forward: TGCCCTCTACAACCAACACA Reverse: GTTGGACAACTGCTCCACCT	277
IL-23	Forward: TGTGCCCCGTATCCAGTGT Reverse: CGGATCCTTTGCAAGCAGAA	81
IL-17F	Forward: CTGAGGCCCAGTGCAGACA Reverse: GCTGAATGGCGACGGAGTT	189
IL-17A	Forward: GCTCCAGAAGGCCCTCAGA Reverse: AGCTTTCCCTCCGCATTGA	187
IFN-γ	Forward: AAAGACAATCAGGCCATCAG Reverse: TGGGTTGTTGACCTCAAACT	129
IL-4	Forward: CATCGGCATTTTGAACGAG Reverse: TTGGAAGCCCTACAGACGAG	120
IL-13	Forward: AGACTCCCCTGTGCAACGGCA Reverse: GGAGACCGTAGTGGGGGCCTT	167
TLR-2	Forward: CGACATCCATCACCTGACTCTTC Reverse: GCCTCGGAATGCCAGCTTCTTC	182
TLR-4	Forward: ACAAGGCATGGCATGGCTTACAC Reverse: TGTCTCCACAGCCACCAGATTCTC	125
Dectin-1	Forward: CCCTCCAAGGCATCCCAAAC Reverse: CCTAGCTGGGAGCAGTGTCT	140

#### Flow cytometry

The lung tissues of mice were cut, transferred to the culture medium, and digested at 37 °C for 40 min using collagenase; lymphocytes were separated using Percoll. The isolated lymphocytes were then added to an RPMI1640 solution containing 10% fetal bovine serum (along with 2 mM glutamine, 100 U/mL penicillin, and 100 mg/mL streptomycin sulfate). Cells at concentrations of 10^6^/mL were incubated with PMA (5 ng/mL) and ionomycin (1 μM) for 2 h in a carbon dioxide incubator, and monensin (2 μM) was added for further culturing for 4 h. After digesting the cells and diluting the cell suspension to 10^6^ cells/mL using sterile PBS solution, 100 μL cell suspension was added into a flow tube. Then, 1 µg each of CD3 and CD4 (Southern Biotech, Birmingham, USA) surface-labeled antibodies were added to the tubes, and the samples were incubated at 4 °C for 30 min. Subsequently, the samples were washed with 2 mL of sterile PBS solution once, and the supernatant was discarded. Then, 1 mL of Foxp3 Fixing/Breaking Membrane Working Solution (eBioscience, San Diego, CA) was added to each tube, and then the sample was mixed and incubated at room temperature for 50 min. After that, 2 mL of 1 × membrane breaking solution was added into each tube, and the sample was centrifuged at 400–600×*g* for 5 min at 15–20 ℃. The supernatant was discarded, and this step was repeated. Subsequently, 1 μg of fluorescence-conjugated antibody was added to detect intracellular antigens (IL4, IFN-γ, and IL-17A) (BD Biosciences) and incubated at room temperature for more than 30 min.

### Statistical analysis

One-way analysis of variance was carried out to assess differences between the infected and control mice at different time points for CFU assay, qRT-PCR, and flow cytometry. Data were visualized using GraphPad Prism 7.0 statistical software, with *p* < 0.05 indicating a statistically significant difference, *p* < 0.01 indicating a highly significant difference, and *p* < 0.001 indicating an extremely significant difference.

### Ethics approval

The methods were carried out in accordance with the approved guidelines. All animal experiments were approved by the Sichuan Agricultural University animal ethics committee.

## Results

### Early *C. cladosporioides* infection induced severe pulmonary inflammatory lesions

**Figure 1:**
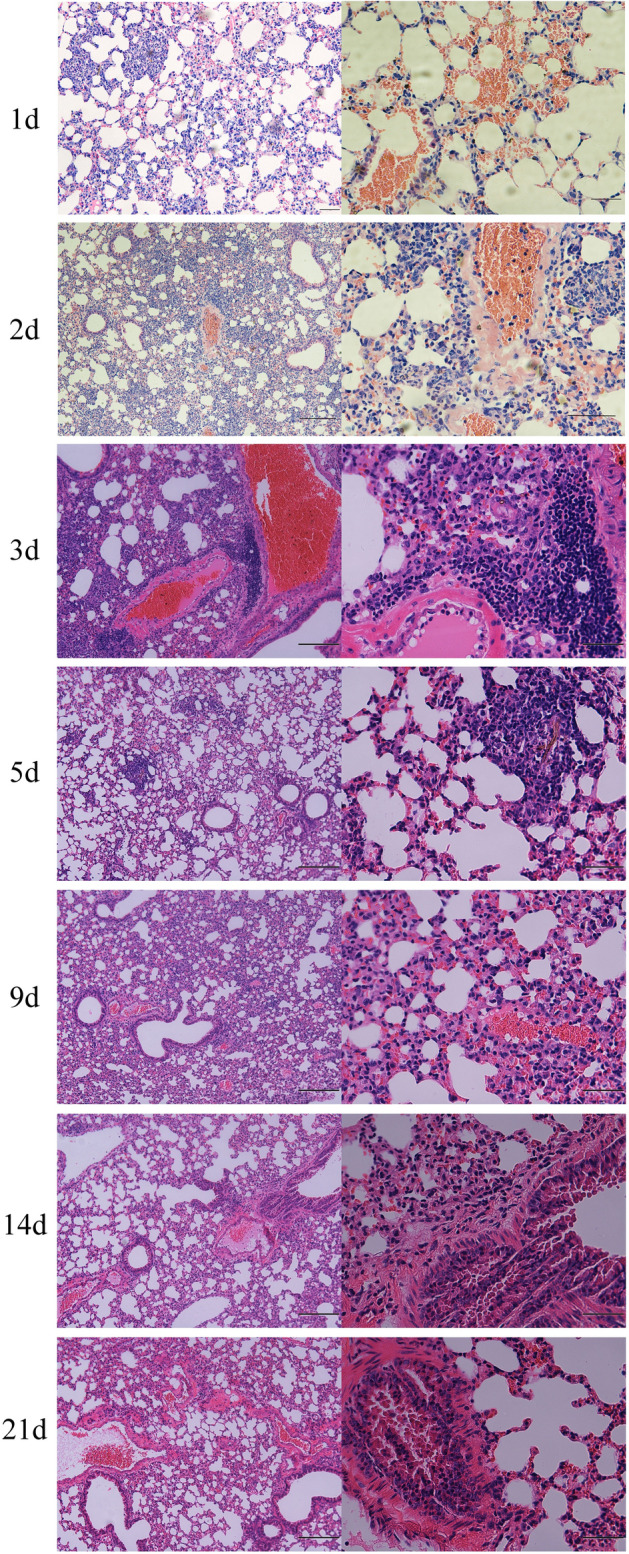
Histological assessment of lung lesions in mice intravenously infected with *Cladosporium cladosporioides*. Severe pulmonary hemorrhage and congestion were observed on days 1, 2, and 3 post-inoculation (PI), and extensive infiltration of inflammatory cells was observed throughout the infection period, especially around veins. Data are representative of three independent experiments (n = 3 mice per group). Scale bars, left-200 μm, right-50 μm.

To histologically assess lesions in mice after infection, pulmonary biopsy samples were stained with H&E (Fig. 1). Extensive infiltration of red blood cells and inflammatory cells was observed in lung tissue after infection. Severe pulmonary hemorrhage and congestion and extensive infiltration of inflammatory cells were observed on day 2 PI. Hemorrhage and hyperemia were gradually relieved with the infection progression; however, extensive inflammatory cell infiltration was observed during an infection period of 21 d. Extensive inflammatory cell infiltration was observed around the blood vessels.

**Figure 2:**
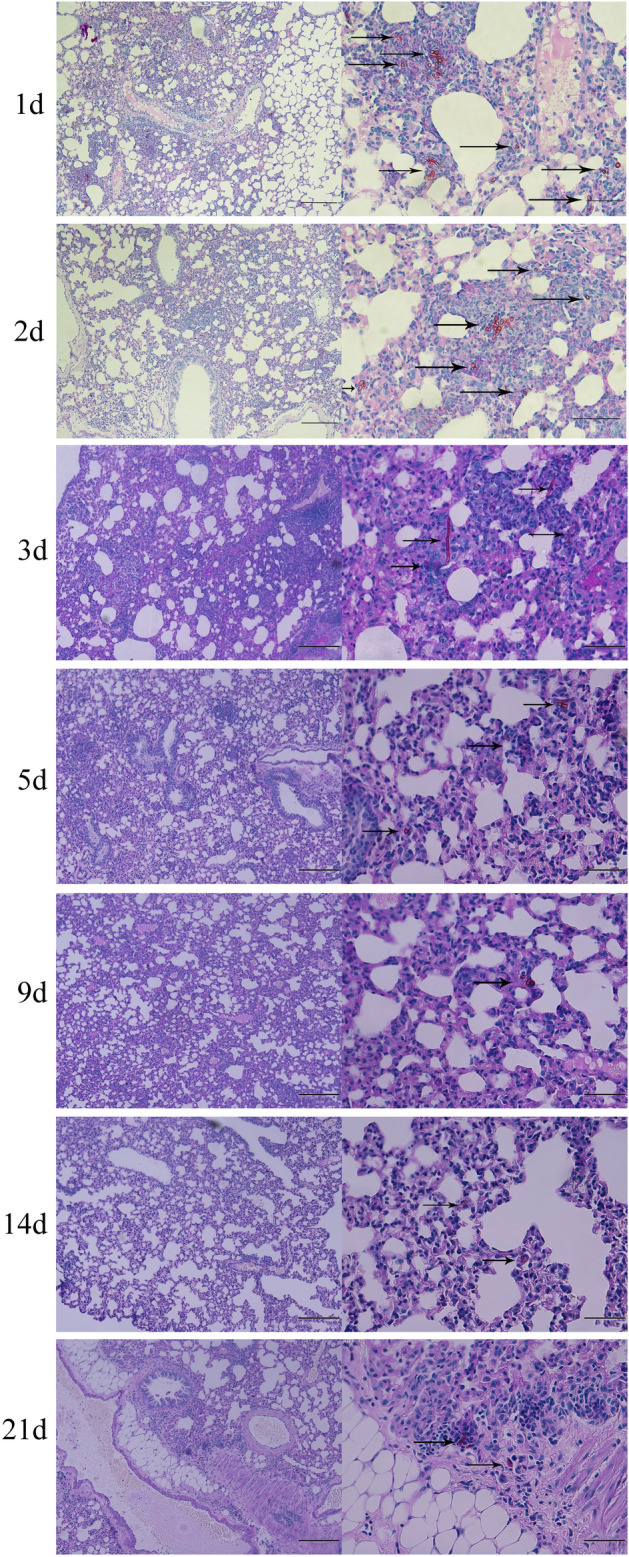
Assessment of the colonization of fungal spores and mycelia in pulmonary structures in mice intravenously infected with *Cladosporium cladosporioides.* A larger number of spores and mycelia colonized the lung tissue on days 1, 2, and 3 post-inoculation (PI) compared to days 5, 9, 14, and 21 PI. The spores and mycelia are indicated with arrows. Data are representative of three independent experiments (n = 3 mice per group). Scale bars, left-200 μm, right-50 μm.

To assess *C. cladosporioides* colonization in pulmonary structures of infected mice, pulmonary biopsy samples were stained with PAS (Fig. 2). Both hyphae and spores were observed in pulmonary structures through the infection period. Numerous spores and hyphae were observed more on days 1, 2, and 3 PI than other time points, primarily concentrated around blood vessels and aggregated. More colonization of fungal hyphae and spores was observed on day 1 PI than on day 2 PI in the lungs. With the infection progression, spore and hyphae colonies were dispersed in pulmonary structures and observed on day 21 PI.

**Figure 3:**
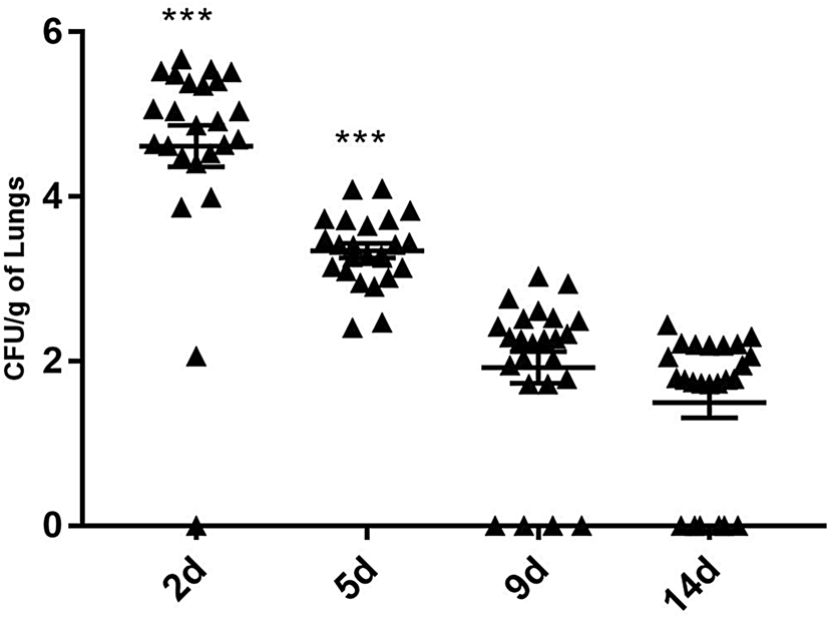
Assessment of the fungal clearance in lungs infected with *Cladosporium cladosporioides*. The fungal burden in lung homogenates were determined on days 2, 5, 9, and 14 post-inoculation. Data are representative of three independent experiments (n = 8 mice per group). *p < 0.05; **p < 0.01; ***p < 0.001.

Furthermore, in the pulmonary CFU assay, fungal colonies were significantly more numerous on days 2 and 5 PI (*p* < 0.001) than at other time points, and fungal colonies were significantly eliminated on day 5 PI (*p* < 0.001), indicating that the *C. cladosporioides* infection was controlled on day 2 PI in lung tissue, while a few fungi colonized the lung tissue on day 14 PI (Fig. 3). These results suggest that spores of *C. cladosporioides* also have a high potential of invading the lungs through pulmonary circulation upon intravenous infection and not only through transtracheal infection.

According to the above results, the number of fungal colonies was most abundant in the first 5 days, causing severe inflammation. As the quantity began to decrease after day 5 PI, the inflammatory response gradually reduced.

### Pulmonary immune cells were activated in response to *C. cladosporioides* infection

**Figure 4:**
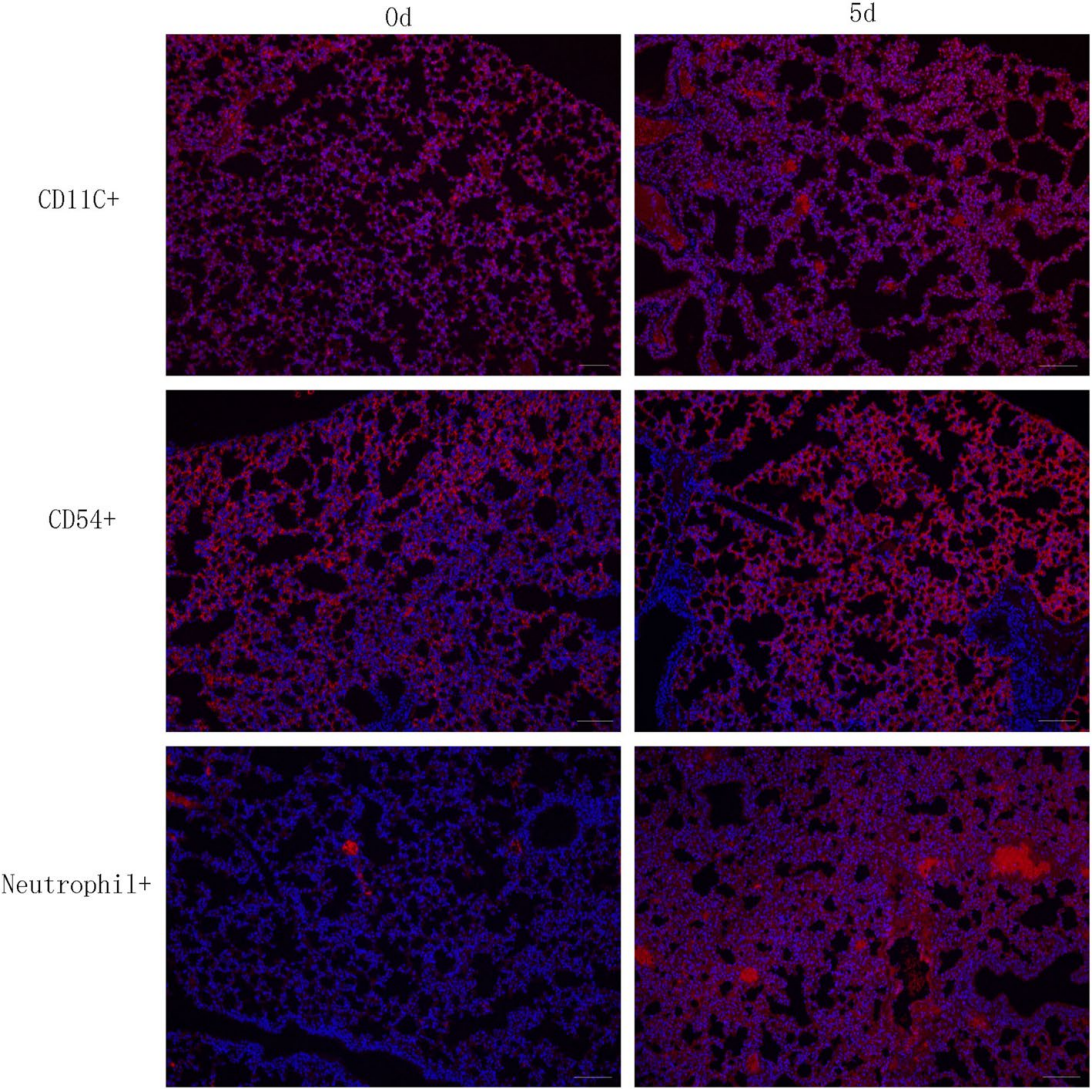
The recruitment of immune cells in the lungs in mice intravenously infected with *Cladosporium cladosporioides.* Numerous polymorphonuclear neutrophils (neutrophil^+^), macrophages, and dendritic cells (CD11C^+^) were recruited in the lung tissue in mice intravenously infected with *Cladosporium cladosporioides*, along with marked CD54 expression (CD54^+^) (red fluorescence increased). Data are representative of three independent experiments (n = 4 mice per group). Scale bars, 200 μm.

Immunofluorescence staining of lung tissue was performed to detect immune cell recruitment on days 0 and 5 PI (Fig. 4). Numerous polymorphonuclear neutrophils (neutrophil^+^), macrophages, and dendritic cells (CD11c^+^) were recruited in the lungs of mice after infection, along with marked CD54 upregulation (CD54^+^). Considering the characteristic features of the lungs and the living environment of mice, a large number of macrophages and dendritic cells were observed in the control group; however, the recruitment of neutrophils, macrophages, and dendritic cells, and CD54 expression in the test groups were markedly greater than those in the control group. These results suggest that forceful pulmonary innate immune responses were induced during a *C. cladosporioides* infection.

### Signaling pathways involved in the immune responses of the lungs after infection

**Figure 5:**
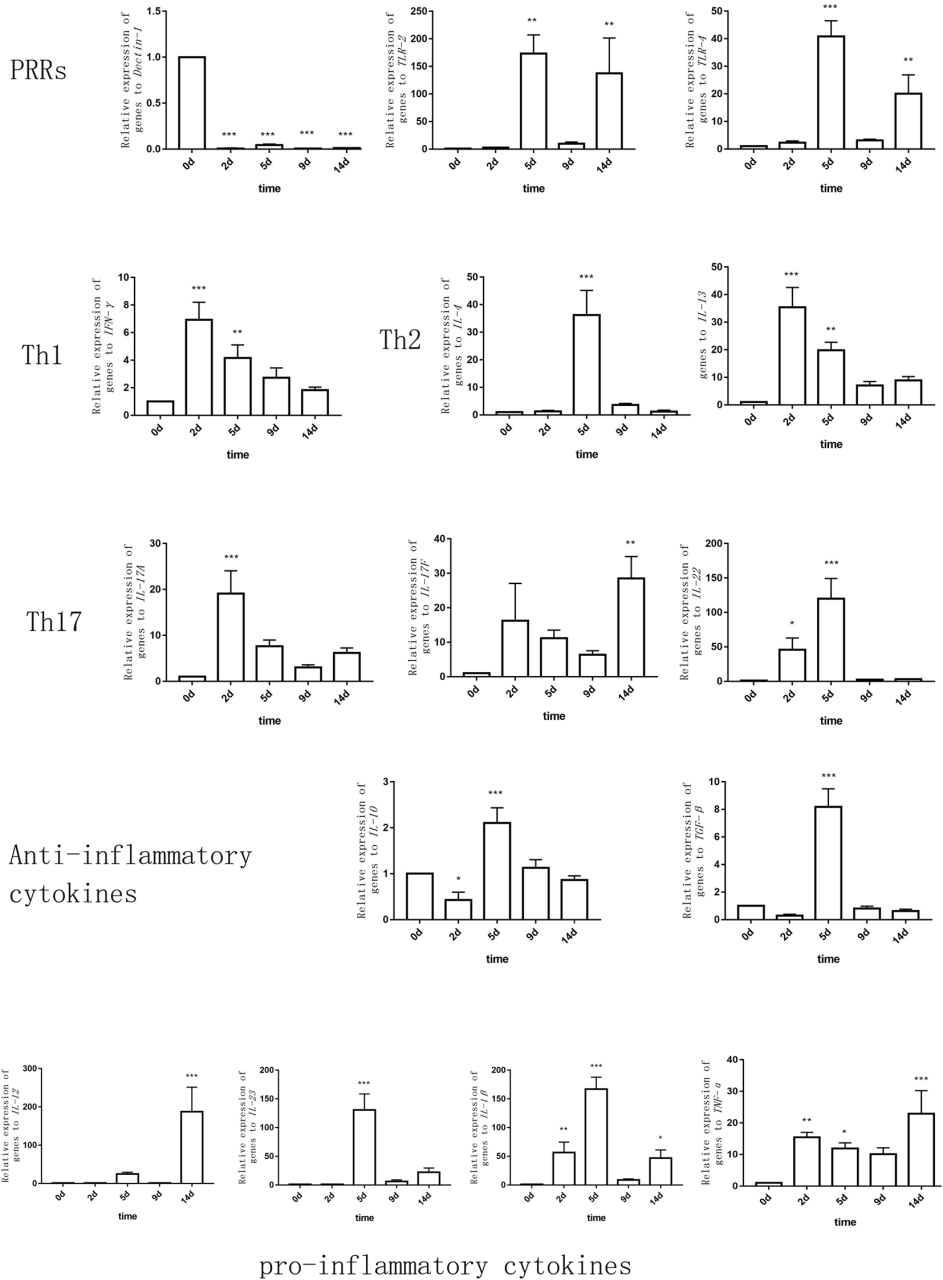
Expression levels of pattern recognition receptor (PRR) and cytokine mRNAs in the lung tissue in the mice intravenously infected with *Cladosporium cladosporioides* on days 0, 2, 5, 9 and 14 post-inoculation (PI). The mRNA expression levels of PRRs and cytokines in the lung tissue of mice in the test groups (days 2, 5, 9, and 14 PI) were measured relative to those of the control group (day 0 PI). Cytokines: PRRs (Dectin-1, TLR-2, TLR-4); Th1 (IFN-γ); Th2 (IL-4, IL-13); Th17 (IL-17A, IL-17F, IL-22); anti-inflammatory cytokines (IL-10, TGF-β); pro-inflammatory cytokines (IL-12, IL-23, IL-1β, TNF-α). TLR-2, TLR-4, IL-1β, IL-10, IL-23, TGF-β, IL-4, and IL-22 mRNAs were significantly upregulated on day 5 PI. IFN-γ, IL-13, and IL-17A were significantly upregulated on day 2 PI. TLR-2, TLR-4, IL-12, TNF-α, and IL-17F were significantly upregulated on day 14 PI. Data are representative of three independent experiments and are expressed as means ± SEMs (n = 8 mice per group).*p < 0.05; **p < 0.01; ***p < 0.001.

The mRNA expression levels of PRRs and cytokines were measured on days 2, 5, 9, and 14 PI relative to the control group. Up- and downregulation of PRR and cytokine mRNAs occurred at some time points after infection (Fig. 5). The increased relative expression levels of IL-12, IL-23, IL-1β, and IL-22 were the highest (over 100), followed by IL-4 and IL-13 (over 30) among all cytokines.

TLR-2/4 mRNA expression levels were significantly higher on days 5 and 14 PI (*p* < 0.01), TLR-2 relative expression folds being greater than those of TLR-4. IL-22 mRNA was gradually upregulated during the infection on days 2 and 5 PI and significantly upregulated on day 5 PI (*p* < 0.001). IL-17A and IL-17F belong to IL-17, and IL-17A mRNA was significantly upregulated on day 2 PI (*p* < 0.001), while IL-17F mRNA was significantly upregulated on day 14 PI (*p* < 0.01). IL-23 mRNA was significantly upregulated on day 5 PI (*p* < 0.001) and plateaued on days 9 and 14 PI (*p* > 0.05). IL-1β mRNA was gradually upregulated during the infection period on days 2 and 5 PI, and days 9 and 14 PI and significantly upregulated on day 5 PI (*p* < 0.001), plateauing on day 14 PI (*p* < 0.05). IL-10 and TGF-β mRNA were significantly upregulated on day 5 PI (*p* < 0.001). TLR-2, TLR-4, IL-22, IL-17A, IL-23, IL-1β, IL-10, and TGF-β mRNAs were significantly upregulated on days 2 and 5 PI. This suggested that fungi were primarily recognized by TLRs and induced acute inflammatory responses and that Th17-associated cytokine genes were highly expressed during these immune responses.

IL-4 mRNA was significantly upregulated on day 5 PI (*p* < 0.001), followed by gradual downregulation. IL-13 was significantly upregulated on day 2 PI (*p* < 0.001) and plateaued on day 5 PI (*p* < 0.05). IL-4 and IL-13 expression trends suggest that Th2-associated cytokine genes are weakly expressed during the immune responses.

TNF-α mRNA was significantly upregulated on day 2 PI (*p* < 0.01) and on day 14 PI (*p* < 0.001). IL-12 was significantly upregulated on day 14 PI (p < 0.001) and plateaued on day 5 PI (*p* > 0.05). With infection progression, numerous cytokines were downregulated relative to the early stages of the infection; however, TLR-2, TLR-4, IL-17F, IL-1β, and TNF-α were significantly upregulated on day 14 PI relative to day 0 PI, suggesting that the rest of the fungi were forcefully eliminated on day 14 PI.

Furthermore, IFN-γ mRNA was significantly upregulated on day 2 PI (*p* < 0.001) and then gradually downregulated. Dectin-1 mRNA was significantly downregulated after infection (*p* < 0.001), suggesting that Dectin-1 could hardly recognize *C. cladosporioides* in the lungs after infection. The significant upregulation of pro-inflammatory cytokines, including IL-4, IL-23, IFN-γ, IL-23, IL-1β, IL-17A, and IL-22, occurred on days 2 and 5 PI. The significant upregulation of anti-inflammatory cytokines IL-10 and TGF-β occurred on day 5 PI. These results indicate that days 2 and 5 mark the period of acute inflammation during a *C. cladosporioides* infection.

**Figure 6:**
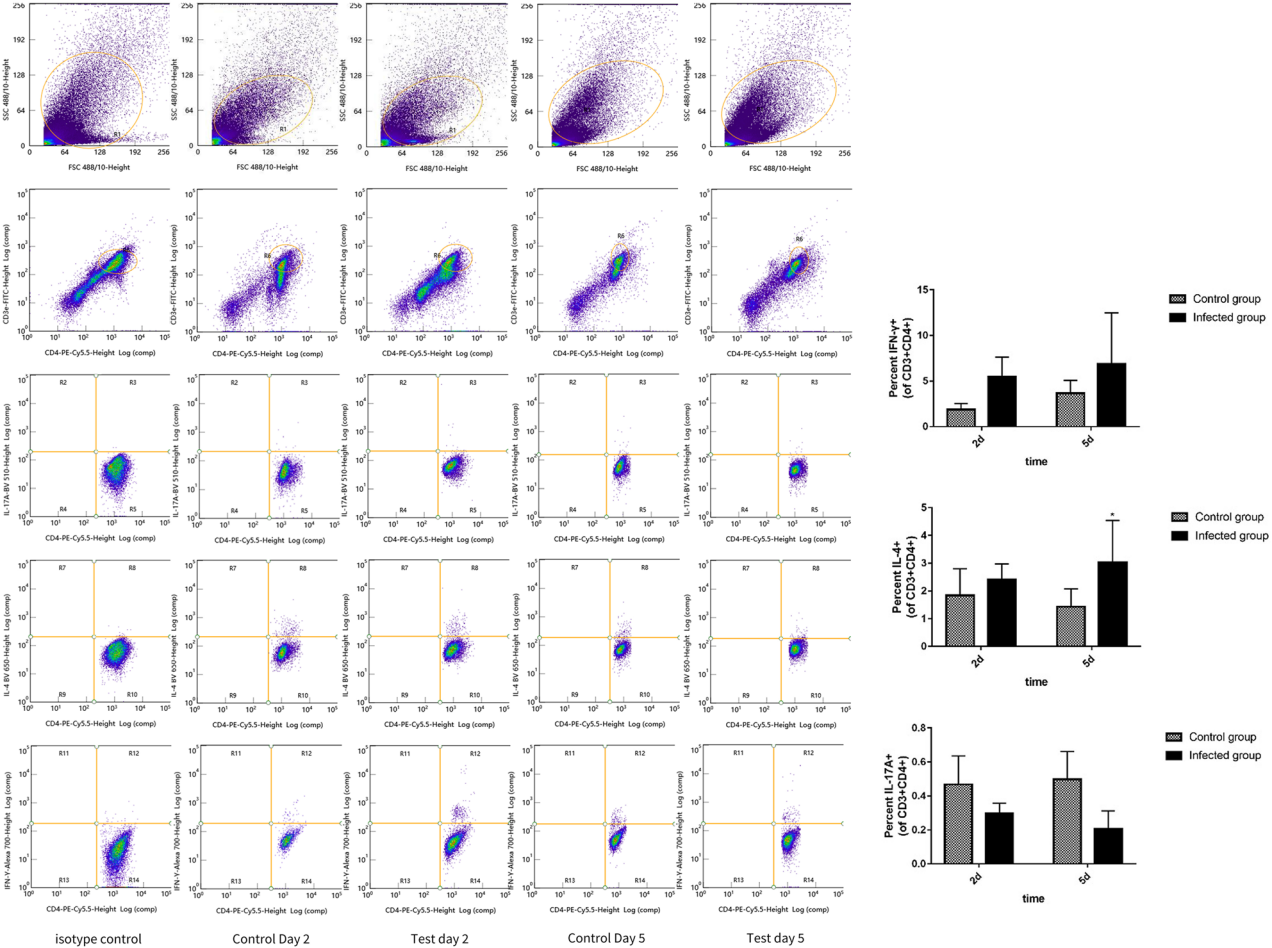
*Cladosporium cladosporioides* induced more Th1 and Th2 cells after infection. We detected Th cells (Th1, Th2, and Th17) with the antibodies IFN-γ, IL-4, and IL-17A, respectively. More Th1 and Th2 cells differentiated after infection. Data are expressed as mean ± SEM (n = 6 mice per group).*p < 0.05; **p < 0.01; ***p < 0.001.

Flow cytometry analysis revealed that Th1 cells increased on days 2 and 5 PI (*p* > 0.05), Th2 cells considerably increased on day 5 PI (*p* < 0.05) than on day 2 PI (*p* > 0.05), and Th17 cells decreased on days 2 and 5 PI (*p* > 0.05) (Fig. 6). These results indicate that the Th2 signaling pathway is involved in immune responses, especially on day 5 PI, followed by the Th1 signaling pathway.

## Discussion

Thus far, studies on *C. cladosporioides* have primarily focused on the pathogenicity of this fungus in the respiratory tract and the lungs upon intratracheal administration, owing to its allergenicity^27,33–35^. Recent studies have reported an increased incidence of superficial and deep infections of *C. cladosporioides*, especially as primary or secondary infections in immunodeficient individuals and animals^27^. Hence, it is important to determine the pulmonary pathological symptoms and immune responses, possibly owing to a potential systemic infection emerging from skin lesions. As such, we conducted this research.

In our experiment, the inoculation of mice with *C. cladosporioides* by intravenous injection caused systemic infections. However, which organ is most seriously infected and which one should be selected for the experiment was unclear. We explored fungal colonization in the heart, liver, spleen, kidney, and lungs with the H&E staining and CFU measurement. The results showed that there were more fungal spore colonies in the lungs and fewer in other organs; hence, we chose the lung as the key organ in our experiment.

The induction of neutrophil extracellular traps is a significant effector function of neutrophils and constitutes an important component of antifungal immune systems, since pathogenic fungi are relatively large^36^, as in the case of *A. fumigates*^37^ and *Cryptococcus neoformans*^38^. Dendritic cells regulate neutrophil distribution in the bone marrow, peripheral organs, and blood^39^. Deep lung tissues contain a well-developed innate immune system that includes alveolar macrophages^40^, which are usually quiescent to prevent alveolar damage and are self-regulated to elicit an appropriate immune response^41^. Interstitial macrophages and dendritic cells expressed high-to-intermediate levels of CD11c^31^. Furthermore, CD54, referred to as intercellular adhesion molecule 1, plays an important role in the formation of functional immune synapses and is essential for modulating the functions of mesenchymal stromal cells via innate inflammatory cells^32^. Herein, numerous macrophages and dendritic cells were recruited and CD54 was expressed in the lungs of control mice, probably because the mice were housed under normal conditions instead of sterile conditions and owing to the specificity of lungs in mice. The recruitment of neutrophils, macrophages, and dendritic cells and CD54 expression together significantly contribute to host innate immune responses.

Total β-glucan levels in the cell wall of *A. versicolor* are lower than those in *C. cladosporioides*; however, its recognition by Dectin-1 is more effective than in *C. cladosporioides* owing to the discernible effectiveness of β-glucan in *A. versicolor* rather than in *C. cladosporioides*, unless the latter is heat-killed^27^. Herein, Dectin-1 mRNA was significantly downregulated on days 2, 5, 9, and 14 PI, suggesting that β-glucan was hardly recognized by Dectin-1 in the lungs, concurrent with previous reports, and *C. cladosporioides* was primarily recognized by TLR-2 and TLR-4 on days 5 and 14 PI. These results suggest that Dectin-1 was rapidly downregulated, probably owing to the low recognizable effectiveness of β-glucan in live *C. cladosporioides,* and the primary pathogenic fungus was *C. cladosporioides* in the lungs after infection, thus further suppressing Dectin-1 expression. It is difficult to recognize live *C. cladosporioides* in the host; hence, this fungus may have invaded deep tissue layers.

The effectiveness of the antifungal response depends on the T cell subset involved in the host immune response against the fungi^42^. The production of IFN-γ preventing recurrent infections and produced locally stimulates the antifungal effect of phagocytes and promotes the homing of Th2 cells to the lungs; therefore, the contribution of IFN-γ in eosinophilic and neutrophilic asthma is also considerable. Here, the increase in IFN-γ and Th1 cells after infection indicated the importance of the Th1 signing pathway in the immune responses of the lungs. Furthermore, cytokines such as IL-4 and IL-13 mediate Th2 immune responses, and Treg cytokines, including IL-10, IFN-γ, and TGF-β, inhibit Th17 differentiation^43^. Herein, the significant high expression of IL-4 and IL-13 and the presence of a high number of IL-4+ cells on days 2 and 5 PI established the vital role of Th2 immune response. IL-17A and IL-22, Th17 cell differentiation products, were significantly upregulated on days 2 and 5 PI, but there was a decrease in IL-17A cells, as detected by flow cytometry. We speculated that it was inhibited by the high expression of IL-4, IFN-γ, and TGF-β, and unsuccessful protein secretion, reflecting the specific pathogenesis of *C. cladosporioides*.

IL-17A regulates IL-17F production, thus limiting its secretion despite the presence of IL-17-stimulating factors such as IL-23^44^. In pulmonary inflammation models, IL-17F induced lesser neutrophil airway infiltration than either IL-17A or the IL-17A/F heterodimer, and IL-17F may be less important than IL-17A in vivo^45,46^. Herein, TLR-2, TLR-4, TNF-α, IL-17F, and IL-12 were markedly expressed on day 14 PI, suggesting that the remaining fungi, which still colonized the lung tissue, induced a marked immune response. Lung tissue displayed a gradual recovery.

The adaptive immune response is induced when the innate immune response is insufficient to eliminate the fungus. In vivo, IL-1 is primarily responsible for acute-phase reactions, including fever and synthesis of acute-phase proteins^47^, such as IL-1β, which is an important pro-inflammatory cytokine that not only activates monocytes, macrophages, and neutrophils but also induces adaptive Th1- and Th17-mediated immune responses^48^. IL-23 belongs to the IL-12 family; however, it regulates IL-17. IL-23 promotes the secretion of IL-17; however, IL-12 inhibits IL-17 secretion in human and mouse immune systems^49,50^. IL-17A and IL-17F prevent infections and elicit a protective response, coordinating infections at mucosal and epithelial surfaces, including the intestines, skin, lungs, and mouth^51^. Furthermore, a characteristic inflammatory response driven by IL-17A is neutrophil accumulation^52^. IL-10 and TGF-β are anti-inflammatory cytokines and promote the secretion of each other in macrophages^53,54^. IL-10 is a key anti-inflammatory cytokine secreted by activated immune cells and inhibits IL-17 expression in Th17 cells and macrophages^55–57^. Herein, IL-10, TGF-β, IL-1β, IL-23, IL-17A, IL-22, TNF-α, IL-4, and IL-13 were significantly upregulated on days 2 and 5 PI, suggesting that days 2 and 5 PI marked the inflammatory peak. Furthermore, large-scale recruitment of neutrophils, macrophages, and dendritic cells, along with CD54 upregulation on day 5 PI, controlled the fungal infection on day 2 PI, suggesting that that days 2 and 5 PI reflect the inflammatory peak in the lungs upon intravenous injection and that numerous fungi are recognized by TLR-2 and TLR-4.

A study reported that asthma induced by intratracheally administered *C. cladosporioides* manifested as a strong Th2 response with IL-4 and IL-13 upregulation, while asthma induced by heat-killed *C. cladosporioides* manifested as a strong Th17 response and a relatively weak Th2 response with IL-17A upregulation^27^. Herein, the relative expression fold change values of IL-4 and IL-13 were next to those of IL-23, IL-1β, and IL-22, suggesting that a higher expression of Th2-associated cytokine genes coincided with the increased percentage of Th2 cells after infection with intravenous injection. This indicated that the Th2 signing pathway is involved in immune responses of the lungs against *C. cladosporioides* administered intravenously. Compared to intratracheal infection-induced Th2 responses, live *C. cladosporioides* spores intravenously injected in healthy mouse induced Th2 and Th17 responses, probably because the spores in lungs and blood circulation inducing the infection were not completely hypersensitive, and thus, may act as normal antigens.

Previous studies on pulmonary infections induced by fungi and other direct allergens (such as house dust mite) have reported that the immune responses in vivo may include Th1, Th17, and Th2 responses, especially a combination of Th2/Th17 responses, which has been reported in serious allergic asthma, along with marked IL-17A and IL-13 expression^33,58^. For example, asthma induced by *A. versicolor* intratracheally manifested as a strong Th17 cell response along with IL-17A, IL-17F, and IL-22 expression, and co-exposure to the house dust mite and *A. versicolor* also resulted in a combination of Th2 and Th17 responses. Th17-associated cytokines influence asthma pathogenesis and severity. For instance, IL-17A contributes to asthma pathogenesis and exerts synergistic effects with IL-13 to induce airway hyper responsiveness^58,59^. Here, live *C. cladosporioides* spores mostly colonized the lungs, and fungal pathogenicity perhaps resulted in allergenicity, although the infection route was different from the intratracheal route and was without severe respiratory symptoms, with the low Th17 cytokines causing no promotion of respiratory symptoms.

In our study, the route of infection and our findings are different from those of previous studies on *C. cladosporioides* and other fungi, considering that the pathogenicity of *C. cladosporioides* results from not only its potential to induce hypersensitivity but also systemic and local infections through its antigens. The Th2 signaling pathway on day 5 PI, mainly, and the Th1 signing pathway, were involved in pulmonary immune responses against *C. cladosporioides.* The pulmonary infection induced via a systemic infection of *C. cladosporioides* potentially results in slight allergic inflammation and less aggressive inflammation via pulmonary phaeohyphomycosis. The living environments of giant pandas in Sichuan, China, with high humidity and an appropriate temperature favoring *C. cladosporioides* proliferation in other animals and humans, more likely contributed to this fungal infection in the environment and the air. *C. cladosporioides* infections in superficial tissue may potentially result in systemic infections through progression to deep-tissue infections.

In conclusion, numerous *C. cladosporioides* cells colonized in the lungs and caused severe inflammation in the lung tissue of mice in this study. During the infection period, the fungus was gradually eliminated, and the host was recovering phase as of day 14 PI. Adaptive immune responses exert synergistic effects during *C. cladosporioides* infection based on innate immune responses. This study provides a basis for not only the immune response and the treatment of diseases occurring through systemic *C. cladosporioides* infections but also the maintenance of the health of giant pandas.
